# Characterization of novel double-reporter strains of *Mycobacterium abscessus* for drug discovery: a study in mScarlet

**DOI:** 10.1128/spectrum.00362-24

**Published:** 2024-08-27

**Authors:** Clara M. Bento, Kevin van Calster, Tatiana Piller, Gabriel S. Oliveira, Linda de Vooght, Davie Cappoen, Paul Cos, M. Salomé Gomes, Tânia Silva

**Affiliations:** 1 i3S—Instituto de Investigação e Inovação e Saúde, Universidade do Porto, Porto, Portugal; 2 IBMC—Instituto de Biologia Celular e Molecular, Universidade do Porto, Porto, Portugal; 3 Programa Doutoral em Biologia Molecular e Celular (MCBiology), Instituto de Ciências Biomédicas Abel Salazar da Universidade do Porto, Porto, Portugal; 4 Laboratory for Microbiology, Parasitology and Hygiene (LMPH), Wilrijk, Belgium; 5 ICBAS—Instituto de Ciências Biomédicas Abel Salazar da Universidade do Porto, Porto, Portugal; University at Albany, Albany, New York, USA

**Keywords:** *Mycobacterium*, drug screening, fluorescence, macrophages, infection models, reporter strains, luminescence, *Galleria mellonella*

## Abstract

*Mycobacterium abscessus* (Mab) is an emerging pathogen that poses a severe health threat, especially in people with cystic fibrosis and other chronic lung diseases. Available drugs are largely ineffective due to an exquisite intrinsic resistance, making Mab infections only comparable to multidrug-resistant tuberculosis. Current treatment is based on lengthy multidrug therapy, complicated by poor outcomes and high rates of treatment failure, recurrence, and mortality. Thus, finding new and more efficient drugs to combat this pathogen is urgent. However, drug discovery efforts targeting Mab have been limited, and traditional drug screening methods are labor-intensive, low-throughput, and do not reflect clinical effectiveness. Therefore, this work aimed to develop a new, efficient, and reliable tool for drug screening against Mab that can be used *in vitro* for identifying hits in a high-throughput manner and *in vivo* to select drug candidates for future clinical trials. We engineered two stable double-reporter strains of Mab capable of emitting strong fluorescent and luminescent signals. This is due to the expression of mScarlet protein and luciferase enzyme or the entire lux operon. Importantly, these strains maintain the same ground characteristics as the non-transformed Mab strain. We show that these new strains can be applied to various setups, from MIC determination in broth cultures and macrophage infection assays to *in vivo* infection (using the *Galleria mellonella* model). Using these strains enhances the potential for high-throughput screening of thousands of compounds in a fast and reliable way.

**IMPORTANCE:**

*Mycobacterium abscessus* (Mab) is currently considered an “incurable nightmare.” Its intrinsic resistance, high toxicity, long duration, and low cure rates of available therapies often lead to the clinical decision not to treat. Moreover, one of the significant drawbacks of anti-Mab drug development is the lack of correlation between *in vitro* susceptibility and clinical efficacy. Most drug screening assays are performed on Mab growing in liquid cultures. But being an intracellular pathogen, inducing granulomas and biofilm formation, the broth culture is far from ideal as *in vitro* drug-testing setup. This study presents new double-reporter Mab strains that allow direct real-time bacterial detection and quantification in a non-invasive way. These strains can be applied to an extensive range of experimental settings, far surpassing the utility of single-reporter bacteria. They can be used in all steps of the pre-clinical anti-Mab drug development pipeline, constituting a highly valuable tool to increase its success.

## INTRODUCTION

Infections by *Mycobacterium abscessus* (Mab) are steadily increasing worldwide and are notoriously difficult to treat. This is primarily due to the bacterium’s exquisite intrinsic resistance to most antibiotics, including first-line anti-mycobacterial drugs and some second-line agents comparable to multidrug-resistant tuberculosis. Current treatment involves lengthy multidrug therapy, often complicated by adverse side effects leading to poor patient compliance (1, 2). Along with the emergence of acquired drug resistance, this leads to an average cure rate of less than 50% (3–5). This low efficacy underscores the need for new and more effective treatments. However, several challenges seriously hamper anti-Mab drug discovery (6–8). First, high-throughput screenings with Mab are not an attractive investment for most laboratories. Due to its high drug resistance, they suffer from lower hit rates compared to more drug-susceptible mycobacterial species, such as *M. tuberculosis* (9–12). Moreover, studies with Mab commonly rely on traditional methods for evaluating *in vitro* antibiotic activity, such as time-, labor-, and resource-consuming colony counting, optical density, or resazurin dye-based methods (9). Second, contrary to other bacterial infections, for which low minimal inhibitory concentrations (MIC) values anticipate successful clinical outcomes, a disappointing lack of correlation is often observed between *in vitro* MICs and *in vivo* efficacy toward Mab (13, 14). Third, all traditional assays are performed under optimal liquid broth conditions, which do not reflect the *in vivo* situation where Mab can grow extracellularly, invade host cells, and/or form biofilms (15). To correct this fundamental flaw, more predictive assays reflecting the different physiological states of Mab, such as macrophage infection assays and biofilm formation, have recently been used in Mab drug discovery pipelines (13, 16). However, those assays still rely on labor-intensive colony counting (17).

Reporter-based assays provide an elegant solution to most of these problems and have a proven track record in high-throughput drug discovery programs with members of the *M. tuberculosis* complex and *M. avium* complex. Remarkably, reporter-based assays in Mab are scarce. Therefore, we aimed to develop a new, efficient, and reliable tool for drug screening against Mab that can be used *in vitro* for high-throughput hit identification and, after that, in animal models to select drug candidates for future clinical trials.

In this study, we engineered new Mab double-reporter strains, which emit luminescence through either a bacterial luciferase encoded by the *LuxABCDE* operon (Mab operon_mScarlet) or the red-shifted derivative of the firefly luciferase (FFrtCO) (Mab FF_mScarlet) in addition to expressing the fluorescent protein mScarlet. We have validated the use of these strains for drug development against Mab, using the luminescent and fluorescent signals as readouts of bacterial load and viability in different setups, from broth cultures to macrophage and *in vivo* infection.

## MATERIALS AND METHODS

### Reagents

Reagents were obtained from Merck Life Science unless stated otherwise.

### Bacterial strains, media, and culture conditions

*Mycobacterium abscessus* ATCC 19977 was grown on Middlebrook 7H10 or 7H11 agar medium containing 0.5% glycerol and 10% Oleic acid-Albumin-Dextrose-Catalase (OADC) (BD Bioscience) or in Middlebrook 7H9 broth (BD Bioscience) containing 0.5% glycerol, 0.05% Tyloxapol, and 10% OADC. When needed, liquid cultures were declumped by passing them through a 10-µm cell strainer (PluriSelect).

For plasmid propagation, the *Escherichia coli* strain DH5α was cultivated using Luria-Bertani (LB) medium.

Both species were grown at 37°C, and liquid cultures were grown with aeration.

When needed for selection purposes, the following antibiotics were added: 100 µg/mL of ampicillin, 25 µg/mL of kanamycin, and zeocin [25 µg/mL for *E. coli*, 50 µg/mL for Mab (Invitrogen)].

### Construction of dual luminescent-fluorescent reporter plasmids

Vectors and suicide plasmids used in this study are listed in Table S1. Primers used for DNA fragment amplification were designed using SnapGene software (from Dotmatics; available at snapgene.com) and are listed in Table S2. A visual representation of the cloning strategy for constructing the double reporter plasmids is shown in Fig. S1. Reagents were obtained from New England Biolabs.

The plasmids were constructed by PCR amplification of specific DNA fragments with Q5 High-Fidelity 2X Master Mix. The resulting PCR products were purified for downstream assembly steps using the Monarch DNA Gel Extraction Kit. Plasmids were constructed using NEBuilder HiFi DNA Assembly Master Mix.

Both reporter constructs were based on the pMV306DIhsp + LuxG13 backbone. This backbone was linearized using the NheI and SpeI restriction sites to exchange the kanamycin resistance cassette with the zeocin resistance cassette (BleoR) from pkm496 (18) and simultaneously introduce the ttsbiB terminator from pML1357 (19). The resulting plasmid was linearized to introduce the Pleft* promoter and mScarlet, both derived from the plasmid L5 attB::Pleft* mScarlet (20) together with ttsbiA terminator from Pml1357 to create the new plasmid pMV306DIhsp + LuxG13+mScarlet.

The codon usage of a red-shifted thermostable variant of firefly luciferase was optimized for *Mycobacterium* species using JCat (21). The codon-optimized fragment with an upstream-optimized mega Shine Dalgarno sequence (megaSD) (22) was synthesized by Genscript.

**Fig 1:**
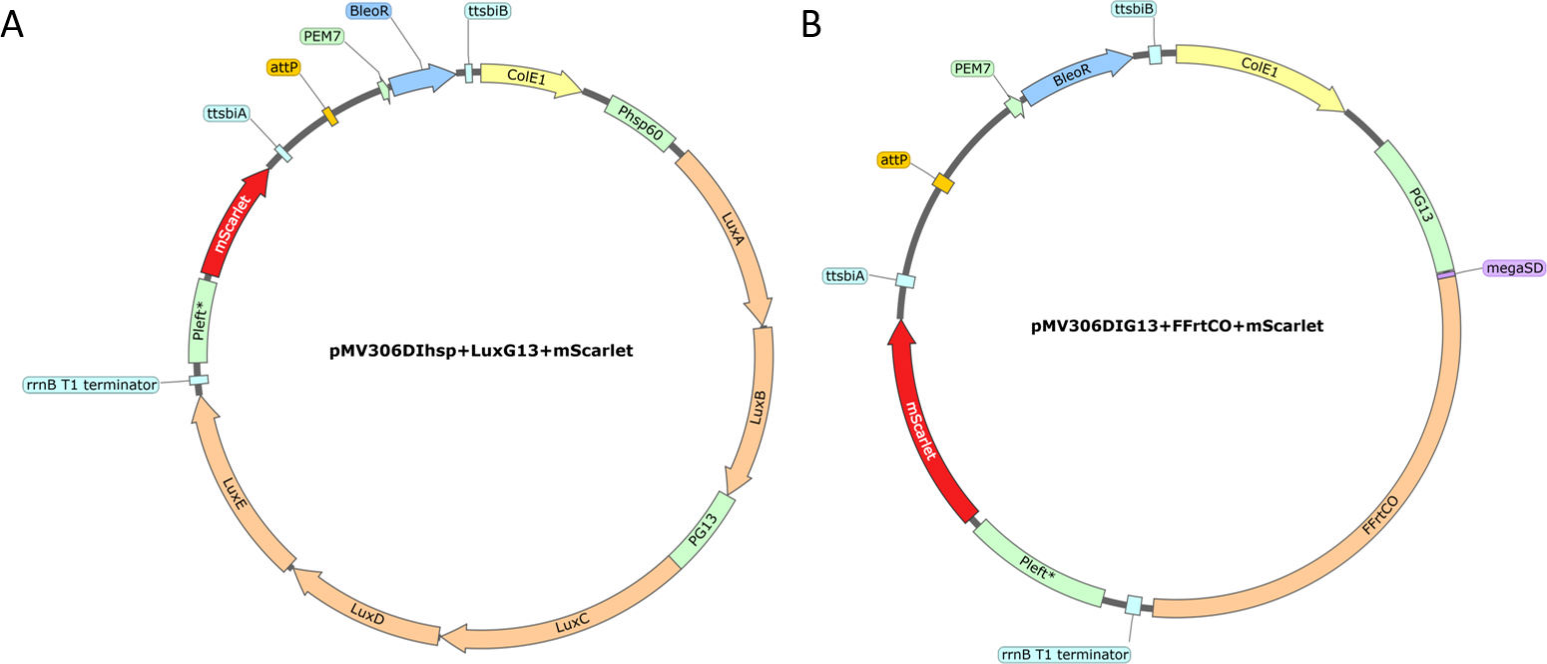
Visualization of the plasmids pMV306DIhsp + LuxG13+mScarlet (**A**) and pMV306DIG13 + FFrtCO + mScarlet (**B**) used to transform Mab WT and generate Mab operon_mScarlet and Mab FF_mScarlet, respectively. The images were obtained using SnapGene software (from Dotmatics; available at snapgene.com). BleoR, zeocin resistance cassette; ttsbiA, bi-directional intrinsic terminator A (19); ttsbiB, bi-directional intrinsic terminator B (19); Pleft*, synthetic promotor element (20); megaSD, optimized mega Shine Dalgarno sequence (22); FFrtCO, red-shifted codon-optimized firefly luciferase; PEM7, EM7 promoter; Phsp60, hsp60 promoter (23); PG13, G13 promoter (24); attP, phage L5 attachment site (25, 26); ColE1, *E. coli* origin of replication (27).

The backbone of the pMV306DIhsp + LuxG13+mScarlet plasmid was linearized via PCR to remove the LuxG13 operon. The PG13 promotor and megaSD-FFrtCO codon were amplified from pMV306DIhsp + LuxG13 and pUC57:FFrtCO codon, respectively. The tree DNA fragments were assembled using HIFI assembly. The final plasmid design is depicted in Fig. 1.

### Generation of Mab double-reporter strain

The double-reporter strains, denoted as Mab operon_mScarlet and Mab FF_mScarlet, were generated by electroporation of the corresponding reporter plasmids (pMV306DIhsp + LuxG13+mScarlet or pMV306DIG13 + FFrtCO + mScarlet) in conjunction with the suicide plasmid pBS-Int (28) into electrocompetent (Mab ATCC 19977) cells using a Bio-Rad GenePulser Xcell electroporator (2,500 V, 25 µF, and 1,000 Ω ). Transformants were selected on 7H11 agar containing zeocin. The resulting strains were validated through whole-genome sequencing.

### Transformation stability by flow cytometry

To evaluate if the mScarlet expression is stable over time, Mab operon_mScarlet, Mab FF_mScarlet, and non-transformed Mab (from now on designated as Mab WT) were grown in 7H9 broth at 37°C, 90 rpm until exponential to stationary phase and passaged 5 times. Each sub-culture was sampled twice for cytometry. Briefly, 0.5 mL of bacterial suspension was collected, centrifuged at maximum speed for 5 min, and fixed with PFA 4% for 15 min. The bacteria were then resuspended in phosphate-buffered saline (PBS) with 2% fetal bovine serum (FBS) (Biowest), 1 mM EDTA, and 0.1% sodium azide and filtered through a 35-µm nylon mesh (Corning). The acquisition was performed in a BD LSRFortessa Cell Analyser (BD Biosciences) using the 610/20 nm filter, and data analysis was performed in the FlowJo software (BD Biosciences).

### Luminescence stability in liquid culture

To address the stability of the luminescent signal, exponential phase cultures of Mab operon_mScarlet and Mab FF_mScarlet were plated in 96-well white plates (Thermo Scientific), and the luminescent signal was acquired in 3-min intervals (integration time = 0.1 s) over 30 min using a Synergy Mx microplate reader and the Gen5 software (BioTek, Agilent Technologies). In the case of Mab FF_mScarlet, IVISbrite D-luciferin potassium salt (PerkinElmer) at different concentrations in H_2_O was added to the wells (10%, vol/vol). Results are expressed as Relative Luminescence Units (RLU), corresponding to the raw values provided by the equipment.

### Growth curves

Mab operon_mScarlet, Mab FF_mScarlet, and Mab WT were grown until the exponential phase, diluted to OD_600_ = 0.05, and incubated at 37°C, 90 rpm for 10 days. Every 24 h, a sample of each bacterial culture was transferred to a 96-well black plate (Thermo Scientific) for fluorescence measurement (*λ*_ex_ = 569 nm; *λ*_em_ = 594 nm) or to a 96-well white plate (Thermo Scientific) for luminescence measurement (integration time = 0.1 s) using a Synergy Mx plate reader and Gen5 software (BioTek). For Mab FF_mScarlet, IVISbrite D-luciferin potassium salt at 0.5 mg/mL was added to the wells (10%, vol/vol) before measuring the luminescence. At the same time, OD_600_ of a sample diluted 1:10 in culture media was measured, and colony-forming units (CFU) were determined by plating the bacteria in 7H10 agar. Results are expressed either as Relative Fluorescence Units (RFU) or RLU, corresponding to the raw values provided by the equipment.

### Pearson correlation between bacterial load measurements

Optical density, fluorescence, and luminescence were plotted against CFU counting, and Pearson correlation was performed using the software Prism9 (GraphPad Software Inc.). The analysis provides the Pearson correlation coefficient (*r*) value, where *r* = 1 means a perfect correlation.

### Mycobacterial susceptibility against conventional antibiotics

The following antibiotics were used: clarithromycin (0.0005–4 µg/mL), amikacin (0.016–32 µg/mL), linezolid (0.016–128 µg/mL), and moxifloxacin (0.016–32 µg/mL). The antimicrobial activity was assessed by broth microdilution, following the Clinical and Laboratory Standards Institute (CLSI) guidelines (29) and as described previously (30). Briefly, Mab operon_mScarlet, Mab FF_mScarlet, and Mab WT were grown until the exponential phase in 7H9 broth, and 1–5 × 10^5^ CFU/mL of bacteria were seeded in 96-well white (Mab operon_mScarlet and Mab FF_mScarlet) or black (Mab WT) plates with increasing antibiotic concentrations. Each condition was tested in duplicate. The plates were incubated at 37°C in a humid atmosphere. After 3 days of incubation, Mab operon_mScarlet and Mab FF_mScarlet viability were assessed by luminescence measurement, as described above. Mab WT viability was assessed by resazurin reduction. Briefly, resazurin at 2.5 mM (PBS) was added to the wells (10%, vol/vol) and incubated for 8 h at 37°C. The fluorescence of resorufin, resulting from the conversion of resazurin by metabolically active cells, was measured at *λ*_ex_ = 530 nm and *λ*_em_ = 590 nm in a Synergy Mx plate reader and Gen5 software (BioTek). The results are expressed as the percentage of luminescent/fluorescent intensity obtained in experimental wells relative to the luminescent/fluorescent intensity obtained in nontreated wells.

### MIC and IC_99_ determination

Each antibiotic’s minimal inhibitory concentration (MIC) was obtained by observing the first well without visible turbidity in each experiment. The results represent the minimum and maximum values (interval), in µg/mL, observed in the different independent experiments. The IC_99_ (antibiotic concentration that inhibits by 99% the mycobacterial viability) values were interpolated by fitting the luminescent (for Mab operon_mScarlet and Mab FF_mScarlet) or fluorescent (for Mab WT) experimental data through a 4PL nonlinear sigmoidal curve using the software Prism9 (GraphPad Software Inc.). The respective 95% confidence intervals of the interpolations were also retrieved.

### Strains suitability for high-throughput drug screening assays by *Z*′-factor determination

Mab operon_mScarlet, Mab FF_mScarlet, and Mab WT were grown to the exponential phase, and 1–5 × 10^5^ CFU/mL of bacteria were seeded in a 96-well white (Mab operon_mScarlet and Mab FF_mScarlet) or black (Mab WT) plate. Thirty wells were incubated with clarithromycin at 2 µg/mL (CLA 2), and the other 30 wells were left untreated (CLA 0). After 3 days of incubation, the luminescent (for Mab operon_mScarlet and Mab FF_mScarlet) or resorufin fluorescent (for Mab WT) signal was measured as described above. The *Z*′-factor was calculated using the formula below:


$$Z^{\prime }\text{-factor }=1-\frac{3\left(\sigma_{CLA2}+\sigma_{CLA0}\right)}{\left|\mu_{CLA2}-\mu_{CLA0}\right|},$$

in which *σ* is the standard deviation and *µ* is the luminescence/fluorescence intensity average.

*Z*′ = 1, ideal assay, allowing for complete discrimination between negative and positive results; 1 > *Z*′ ≥ 0.5 excellent; 0.5 > *Z*′ > 0, marginal; *Z*′ = 0, nominal; *Z*′ < 0, unacceptable (31).

### Infection of bone marrow-derived macrophages and intracellular antibiotic activity

Macrophages were derived from the bone marrow of male C57BL/6 mice bred at the i3S animal facility as described previously (32). Macrophages in 96-well PhenoPlate microplates (PerkinElmer) were infected with Mab operon_mScarlet, Mab FF_mScarlet, or Mab WT (MOI = 1) and treated with clarithromycin at 2 and 10 µg/mL, moxifloxacin at 2 and 8 µg/mL, or rifampicin at 8 and 16 µg/mL. Antibiotics’ working concentrations were not cytotoxic toward the macrophages. Each condition was tested in triplicate. The intracellular growth of the three Mab strains was evaluated on the day of infection and 2 days after infection by CFU assay. For Mab operon_mScarlet and Mab FF_mScarlet, the intramacrophagic bacterial load was also assessed by fluorescence microscopy. Briefly, 2 days after infection, the infected cells were fixed with PFA 4% for 10 min, permeabilized with PBS + Triton 0.1% for 15 min, and stained with DAPI and HCS CellMask Deep Red (H32721, Invitrogen) for 30 min. The plates were screened in the automated high-content confocal microscope Opera Phenix Plus (PerkinElmer), and the data were analyzed using Harmony High-Content Imaging and Analysis Software (PerkinElmer) with mScarlet fluorescent signal being used as a measure of bacterial load. The fluorescence microscopy images of the stained infected macrophages were acquired using the same equipment and analyzed with ImageJ/Fiji software. Statistical analysis was performed using the software Prism9 (GraphPad Software Inc.), using a two-way ANOVA with Šidák’s multiple comparisons test.

### Galleria mellonella larvae infection

*Galleria mellonella* larvae were purchased from a local shop, Anaconda Reptiles (Kontich, Belgium), and stored in a box filled with wood chips at 4°C until infection. The protocol followed for the larvae infection was adapted from Meir et al. (33). Briefly, 13 larvae (200 mg average) were injected in the last left proleg with 5 × 10^4^ CFU in a total volume of 10 µL by a 31G needle using a Hamilton syringe. After injection, the larvae were incubated at 37°C, and infection was assessed every 24 h. Larvae entering the pupating process were excluded from the experiment.

### Bioluminescent imaging

Mab operon_mScarlet, Mab FF_mScarlet, and Mab WT were grown to the stationary phase in 7H9 broth and serially diluted 1:2 in a 96-well white plate. Luminescence was measured as described above using the IVIS Lumina III *In vivo* Imaging System (PerkinElmer) after an exposition time of 0.5 s. The luminescent signal was analyzed with the Living Image v4.3.1 software and quantified in radiance. Imaging of the larvae infected with Mab FF_mScarlet was performed 1–10 min after injection of 0.05 µg/g D-luciferin (Firefly Luciferin Potassium Salt; AAT Bioquest) using the IVIS Spectrum *In vivo* Imaging system (PerkinElmer). D-luciferin was administered in the last left proleg of the larvae in a volume ranging between 10 and 20 µL by a 31G needle using a Hamilton syringe. After imaging, the luminescent signal of each larva was analyzed with the Living Image v4.3.1 software and quantified as relative luminescence units (RLU).

## RESULTS

### Construction of dual luminescent-fluorescent reporter plasmids

This work aimed to develop double-reporter Mab strains by designing reporter plasmids that would (i) allow for stable integration of the expression cassettes, (ii) have a dual luminescent-fluorescent readout, and (iii) prevent transcriptional interference. To achieve this, two plasmid systems were created (Fig. 1) expressing either the autoluminescent bacterial luciferase or an improved firefly luciferase, each combined with the fluorescent protein mScarlet.

Both plasmids (Fig. 1) contain the gene for the red fluorescent protein mScarlet combined with the promoter Pleft* and a zeocin resistance marker (BleoR). To obtain Mab operon_mScarlet (Fig. 1A), we selected a *luxABCDE* operon modified for expression in Gram-positive bacteria. The *luxAB* genes, encoding bacterial luciferase, were placed under the control of Phsp60, while the *luxCDE* genes, responsible for synthesizing a long-chain aldehyde substrate, were regulated by the PG13 promoter. For Mab FF_mScarlet (Fig. 1B), we chose an improved variant of the firefly luciferase (FFrtCO) with increased bioluminescence intensity and a red-shifted emission spectrum due to several amino acid mutations (Ile423Leu, Asp436Gly, Leu530Arg, Ser284Thr, Thr214Ala, Ala215Leu, Ile232Ala, Phe295Leu, and Glu354Lys). The DNA fragment was further codon-optimized, increasing the guanine-cytosine (GC) content from 58% to 65%, and was paired with an improved mega Shine Dalgarno sequence (megaSD) under the PG13 promotor control.

The laboratory type strain Mab ATCC 19977 was transformed by electroporation with either pMV306DIhsp + LuxG13+mScarlet or pMV306DIG13 + FFrtCO + mScarlet, successfully generating the double-reporter strains Mab operon_mScarlet and Mab FF_mScarlet, respectively.

### Fluorescence and luminescence are stable

**Fig 2:**
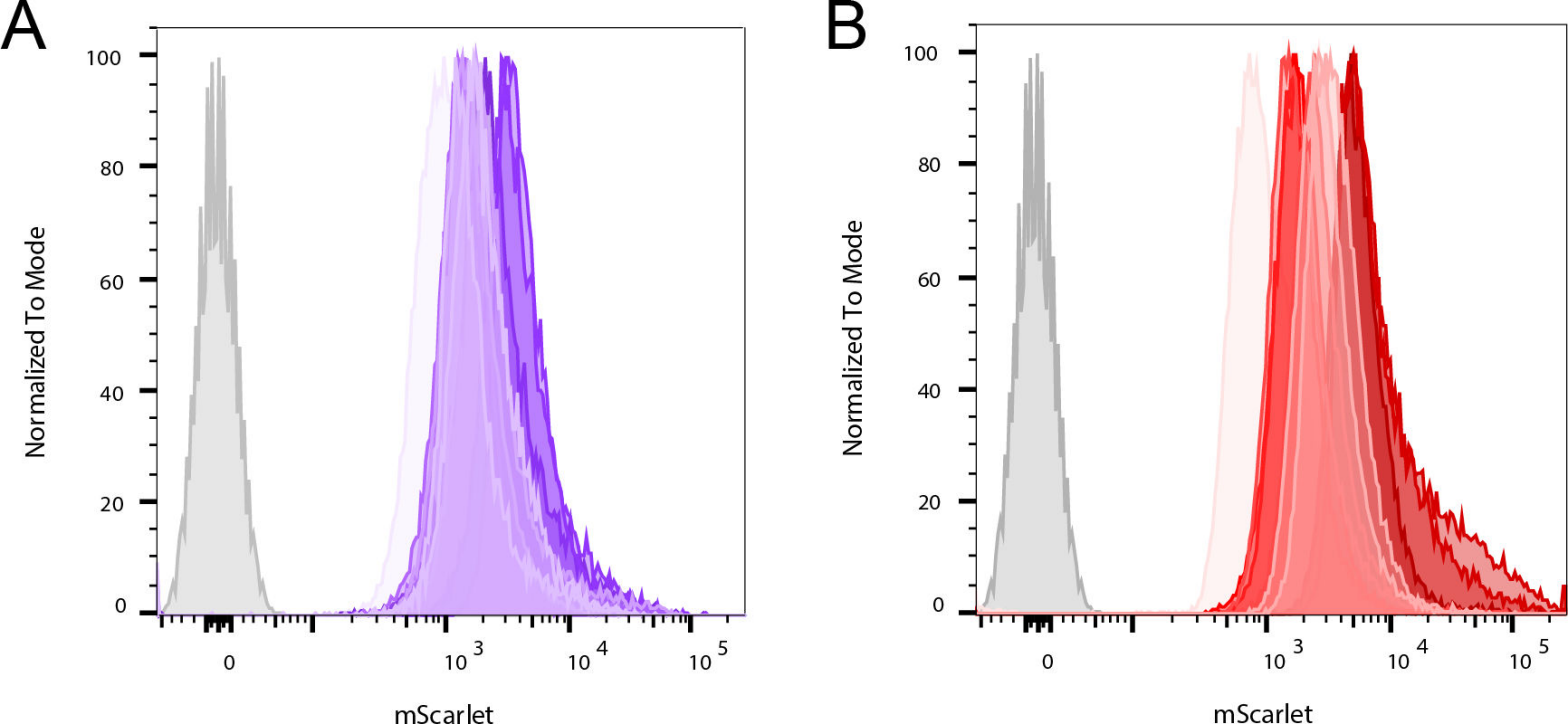
Expression of the mScarlet protein in liquid cultures, as evaluated by flow cytometry, of (**A**) Mab operon_mScarlet or Mab FF_mScarlet (**B**). The three strains were cultured in 7H9 broth, passaged five times, and sampled twice for cytometry in each sub-culture. Each subculture is depicted in a different color, going from a lighter to a darker tone as the number of passages increases. The samples were analyzed in a BD LSRFortessa Cell Analyser (BD Biosciences) using the 610/20 nm filter, and the histograms were obtained with FlowJo software. The grey peak corresponds to ab WT.

After the generation of the double-reporter strains, the next step was to evaluate if the transformation was stable. Specifically, if the double-reporter Mab strains maintain their fluorescence throughout time when sub-culturing in a medium without the selection antibiotic (zeocin). To achieve this, mScarlet expression was evaluated by flow cytometry (Fig. 2). The histograms show a clear difference between the fluorescence signal emitted by samples of Mab operon_mScarlet (Fig. 2A) or Mab FF_mScarlet (Fig. 2B) compared to the non-transformed strain, here designated Mab WT. These results show that even after five passages without selective pressure from zeocin, the expression of mScarlet is maintained. We can, thus, presume that the expression of the luminescence genes is also maintained over time.

Regarding luminescence, the strain Mab FF_mScarlet only expresses the enzyme luciferase. For luminescence to occur, the enzyme’s substrate, D-luciferin, must be added at the desired time-point. Thus, the concentration of D-luciferin and the incubation time needed to obtain the maximum value were assessed in two Mab FF_mScarlet cultures differing in the number of cells.

We observed that 0.5 mg/mL of D-luciferin produces an intense and stable luminescent signal for up to 30 min, and this stability was independent of the number of bacteria in the culture (Fig. S2B). Therefore, this was the concentration selected to proceed with the antibiotic susceptibility assays, and 10 min was the period chosen to measure the luminescent signal after adding D-luciferin. Similarly, the autoluminescence of two liquid cultures of Mab operon_mScarlet at different optical densities was also measured for 30 min (Fig. S2A), confirming the stability of the luminescent signal without adding a substrate.

### The double-reporter Mab strains grow similarly to the non-transformed strain, and their fluorescence and luminescence correlate with CFU counting

**Fig 3:**
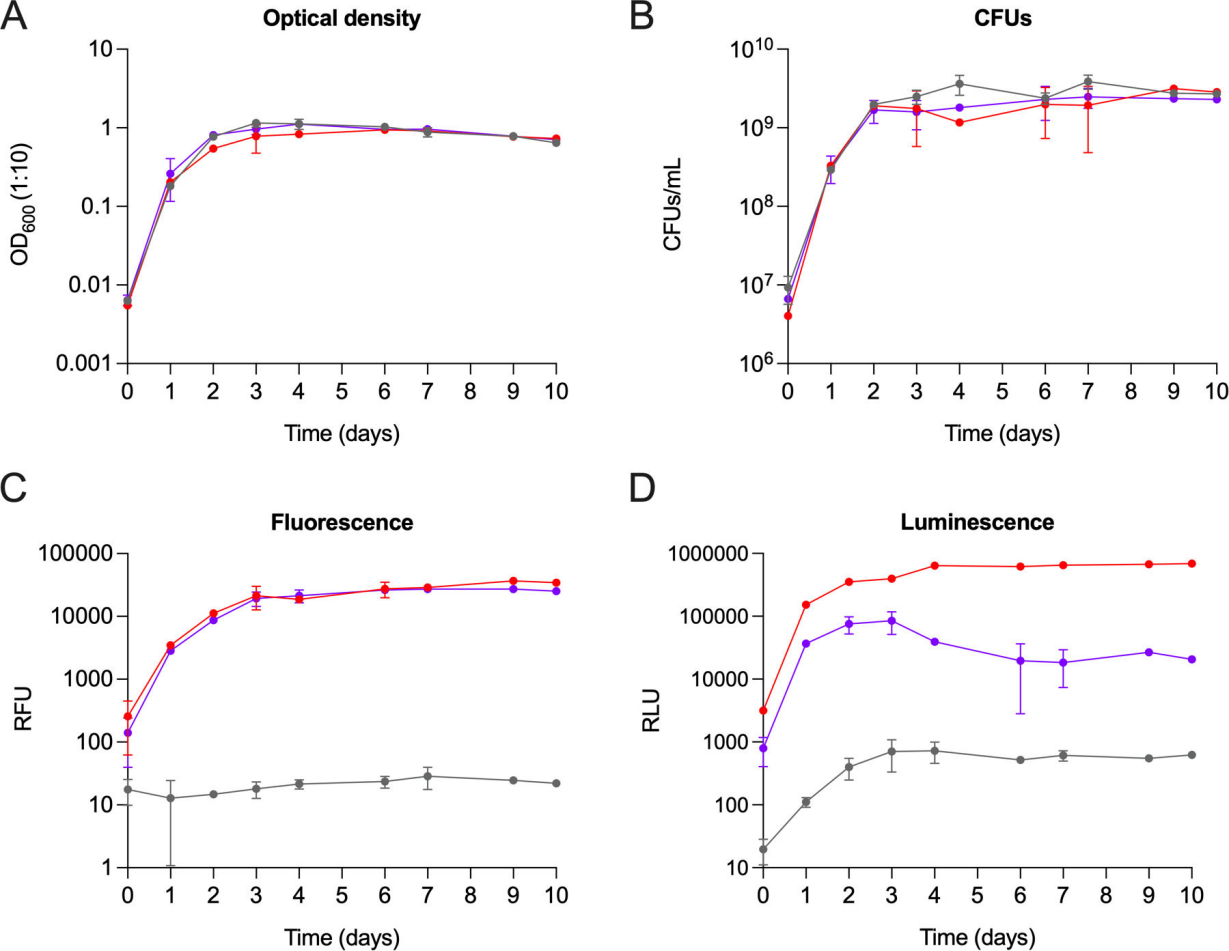
Growth curves of the double-reporter Mab strains and the non-transformed Mab strain. Liquid cultures of Mab WT (gray), Mab operon_mScarlet (purple), and Mab FF_mScarlet (red) were incubated at 37°C, 90 rpm, for 10 days. In each time-point, samples were taken for (**A**) OD_600_ (1:10), (**B**) CFU counting, (**C**) fluorescence at 569/594 nm, and (**D**) luminescence. Fluorescence and luminescence were read in a Synergy Mx plate reader. The symbols represent the average ± standard deviations of two to three independent experiments. RFU, relative fluorescence units; RLU, relative luminescence units.

The growth profiles of the double-reporter Mab strains were compared with Mab WT by simultaneously measuring optical density (OD_600_), fluorescence, luminescence, and CFU. The optical density and CFU results show that the double-reporter strains grew similarly to Mab WT (Fig. 3A and B). The fluorescent and luminescent signals were similar between the two transformed strains, except that Mab operon_mScarlet had a lower luminescent signal when compared to Mab FF_mScarlet; after day 4, there was a slight decline in luminescence intensity (Fig. 3D).

**Fig 4:**
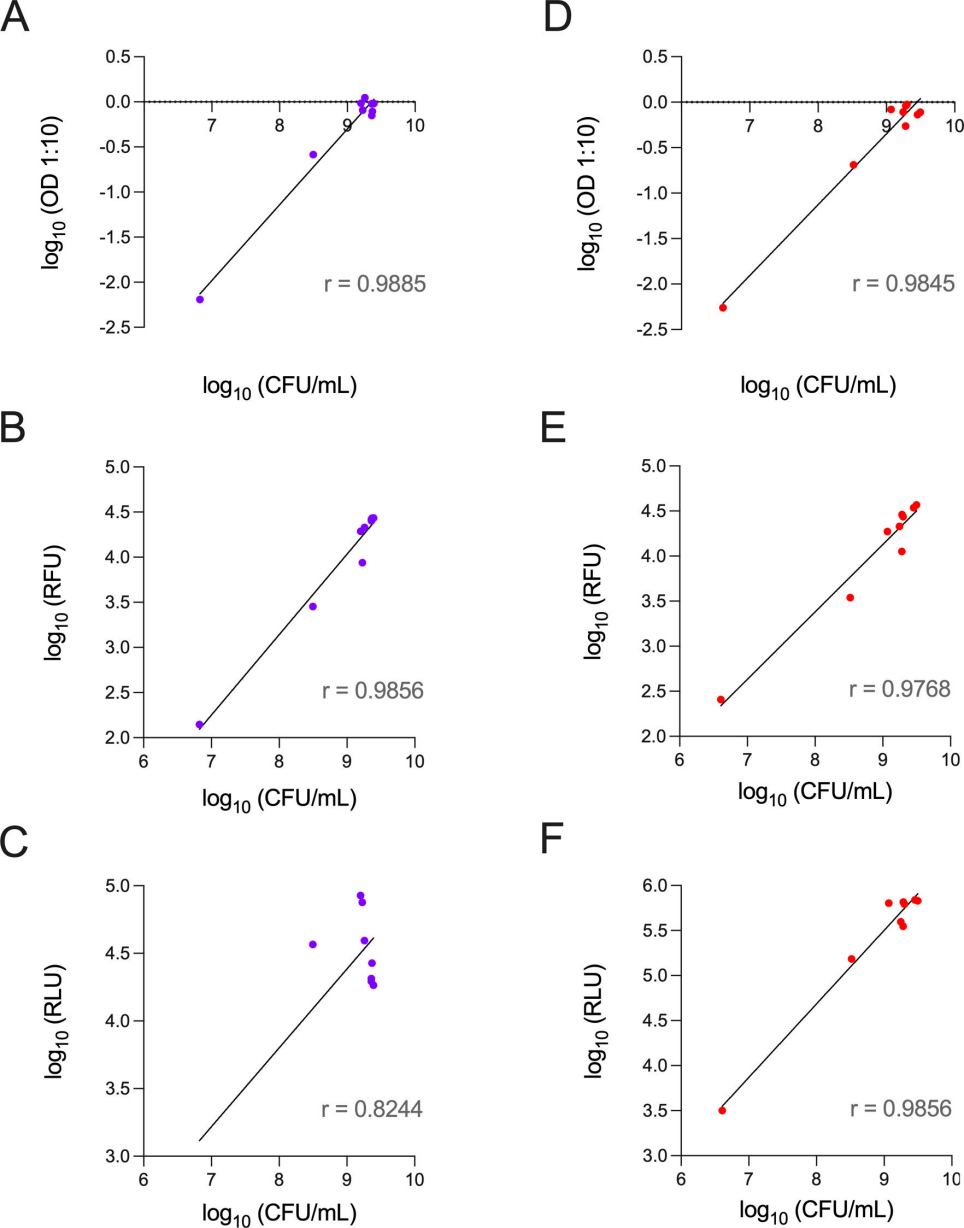
Pearson correlation between CFU counting and (**A and D**) OD_600_ (1:10), (**B and E**) fluorescence, and (**C and F**) luminescence of the double-reporter Mab strains. (**A–C**) Mab operon_mScarlet; (**D–F**) Mab FF_mScarlet. The symbols represent the average of the experiments from Fig. 3. RFU, relative fluorescence units; RLU, relative luminescence units. The lines represent a nonlinear fit, and *r* is the Pearson correlation coefficient calculated with GraphPad Prism 9.

Moreover, by plotting optical density, fluorescence, and luminescence parameters against CFU, it is possible to conclude that the fluorescent and luminescent signals emitted by the double-reporter Mab strains, as well as the OD_600_, have an excellent correlation with the CFU counting (Fig. 4). In terms of luminescence, the *r* value for Mab operon_mScarlet indicates a good correlation although it is not as good as the Mab FF_mScarlet results. This lower correlation is associated with decreased luminescent intensity after day 4 (Fig. 3D), which is not in line with the CFU counting in that period (Fig. 3A). Notably, the fluorescent and luminescent signals were similar in the presence of zeocin (data not shown) or in the absence of zeocin (Fig. 3). Overall, the data above show the suitability of these strains for drug screening.

### The double-reporter Mab strains have similar susceptibility to antibiotics as the non-transformed strain

**Fig 5:**
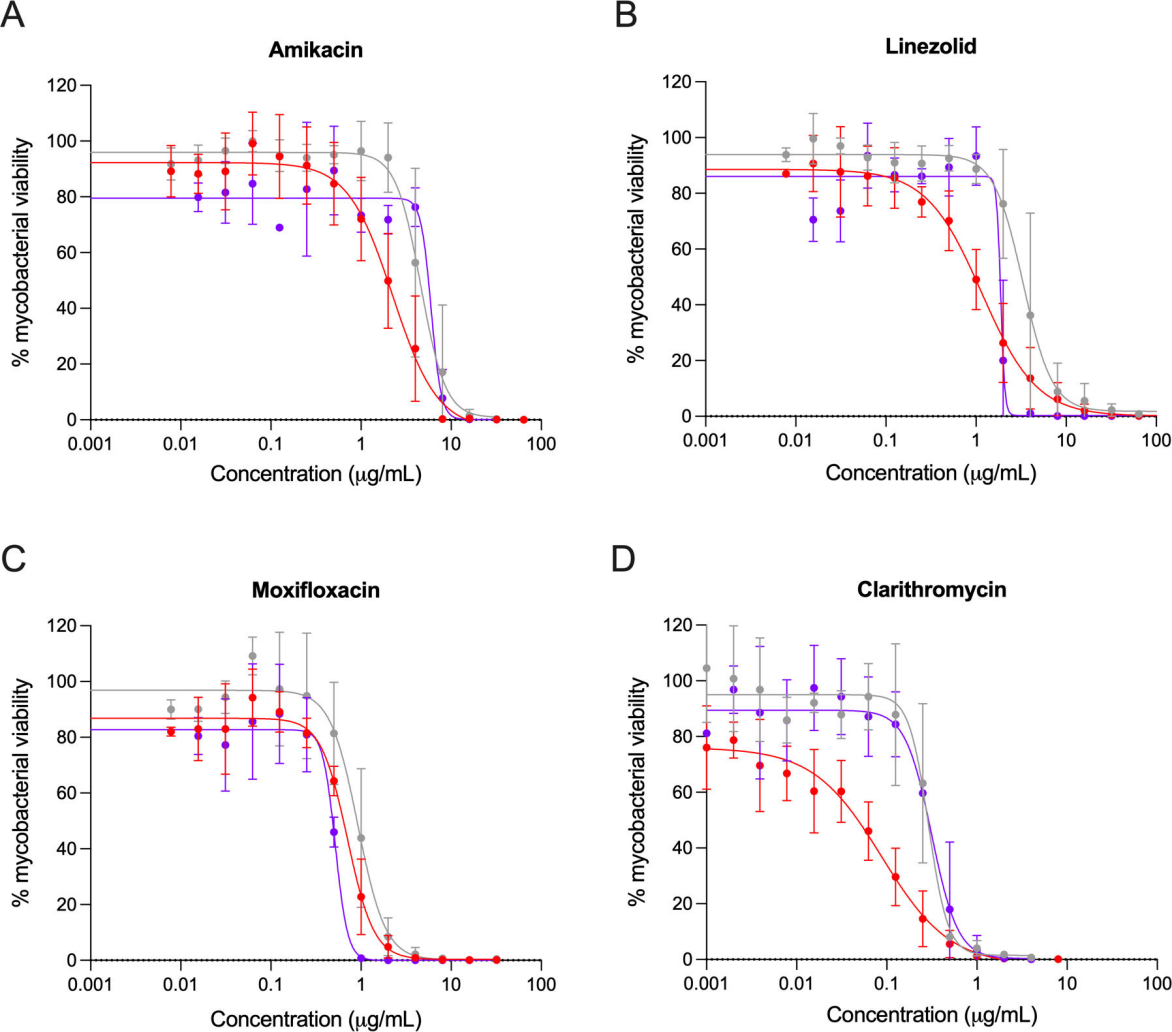
Antibiotic susceptibility profile of Mab operon_mScarlet (purple), Mab FF_mScarlet (red), and Mab WT (gray). Each strain was incubated with increasing concentrations of (**A**) amikacin, (**B**) linezolid, (**C**) moxifloxacin, and (**D**) clarithromycin for 3 days. The symbols represent the average ± standard deviations of three to seven independent experiments, presented as percentages of viable mycobacteria relative to the non-treated mycobacteria, measured by luminescence for Mab operon_mScarlet and Mab FF_mScarlet and by resorufin fluorescence for Mab WT. The lines represent the nonlinear regression (4PL) obtained in the GraphPad Prism 9 for each condition.

To address if the transformation, and specifically the insertion of a resistance gene, altered the Mab susceptibility to conventional antibiotics used in the clinic, the three Mab strains were incubated with increasing concentrations of amikacin, linezolid, moxifloxacin, and clarithromycin for 3 days at 37°C. The mycobacterial viability was evaluated by measuring the luminescent signal of the double-reporter strains or by resazurin assay for Mab WT (Fig. 5).

Overall, the antibiotic susceptibility profile of the double-reporter Mab strains is similar to Mab WT, as reflected in the dose-response curves depicted in Fig. 5. That is corroborated by comparing the values of the minimum inhibitory concentration (MIC) obtained by visual observation and the concentration of the antibiotic that inhibits by 99% the mycobacterial viability (IC_99_) calculated with the luminescence or fluorescence data (Table 1).

**TABLE 1:** Activity of amikacin, linezolid, moxifloxacin, and clarithromycin against liquid cultures of the double-reporter Mab strains and Mab WT

	Mab operon_mScarlet		Mab FF_mScarlet		Mab WT	
Antibiotic	MIC interval^*a*^ (μg/mL)	IC_99_ (μg/mL)^*b*^	MIC interval^*a*^ (μg/mL)	IC_99_ (μg/mL)^*b*^	MIC interval ^*a*^ (μg/mL)	IC_99_ (μg/mL)^*b*^
Amikacin	[8, 32]	10.71 (5.60-n.c.)	[8, 32]	12.96 (7.99-n.c.)	[8, 16]	31.42 (10.03-n.c.)
Linezolid	[2, 8]	2.41 (n.c.)	[4, 32]	28.67 (10.15-n.c.)	[4, 32]	n.c.
Moxifloxacin	[2, 4]	1.04 (0.56-n.c.)	[2, 8]	3.64 (1.98- n.c.)	[2, 8]	4.91 (2.17-n.c.)
Clarithromycin	[0.5, 1]	1.58 (0.57-n.c.)	[0.5, 1]	1.36 (0.63-n.c.)	[0.5, 1]	n.c.

^*a*^ MIC: minimum inhibitory concentration, the first well without visible turbidity, is represented as an interval between the lowest and highest concentration detected in three to seven independent experiments.

^*b*^ IC_99_ (antibiotic concentration that inhibits by 99% the mycobacterial viability) was measured by luminescence for Mab operon_mScarlet and Mab FF_mScarlet and by resorufin fluorescence for Mab WT. The IC_99_ values were obtained by interpolation of nonlinear regression (4PL) of the experimental data obtained in the GraphPad Prism 9. The parenthesis values represent the interpolation’s 95% confidence interval. n.c., not possible to calculate.

Conventionally, the potency of an antibiotic is measured by determination of the MIC value—the minimum antibiotic concentration for which no growth of bacteria is detected—through visual inspection of a liquid culture. This evaluation of antibiotic activity is, however, prone to error due to the subjectivity of the reading. Using fluorescence or luminescence as measures of bacteria viability allows the calculation of IC_99_ (as a *proxi* to complete growth inhibition) from dose-response curves. Table 1 shows that the IC_99_ calculated from luminescence (double-reporter Mab strains) correlates better with the MIC values for each antibiotic than the IC_99_ calculated from resazurin/resorufin fluorescence (Mab WT). Therefore, luminescence is more sensitive than the resazurin assay to evaluate antibiotics’ activity, especially for detecting low quantities of bacteria. The MIC and IC_99_ values found for the double reporter strains did not significantly differ from those of Mab WT, except for an apparent discrete increase in susceptibility to linezolid by Mab operon_mScarlet.

In high-throughput drug screening, one way to evaluate the quality of the assay is by calculating the *Z*′-factor. The *Z′*-factor describes how well separated the positive and negative controls are and indicates the likelihood of false positives or negatives. Thus, we have determined the *Z′*-factor for all Mab strains using 2 µg/mL of clarithromycin as the positive control (low quantity of bacteria) and non-treated bacteria as the negative control Table 2.

**TABLE 2:** *Z′*-factor of a clarithromycin susceptibility assay against the double-reporter Mab strains and the non-transformed Mab strain

	Clarithromycin at 2 µg/mL		Clarithromycin at 0 µg/mL		
Strain	Luminescent intensity average (RLU)^*a*^	Standard deviation	Luminescent intensity average (RLU)^*a*^	Standard deviation	Z’-factor^*b*^
Mab operon_mScarlet	712.73	252.76	29,178.07	1,627.39	0.80
Mab FF_mScarlet	3725.20	299.56	63009.43	3808.39	0.79

	Resorufin intensity average (RFU)^*a*^	Standard deviation	Resorufin intensity average (RFU)^*a*^	Standard deviation	Z’-factor^*b*^
Mab WT	9753.27	1055.66	56584.93	4800.34	0.62

^*a*^ Average of luminescence (for the transformed strains) or resorufin fluorescence (for the non-transformed strain) intensity values of 30 wells treated or not with clarithromycin.

^*b*^ *Z′*-factor can be interpreted as follows: 1 > *Z′* ≥ 0.5 excellent.

As the calculated *Z′*-factors for all Mab strains are between 0.5 and 1, the assay is considered excellent and can be used in a high-throughput context. Besides, the *Z*′-factor value is higher for the double-reporter Mab strains than for Mab WT, which supports using these new strains’ luminescence as a drug screening tool instead of conventional methods like the resazurin assay.

### The double-reporter Mab strains can infect mammalian host cells, and the intracellular bacterial load can be assessed by fluorescence

**Fig 6:**
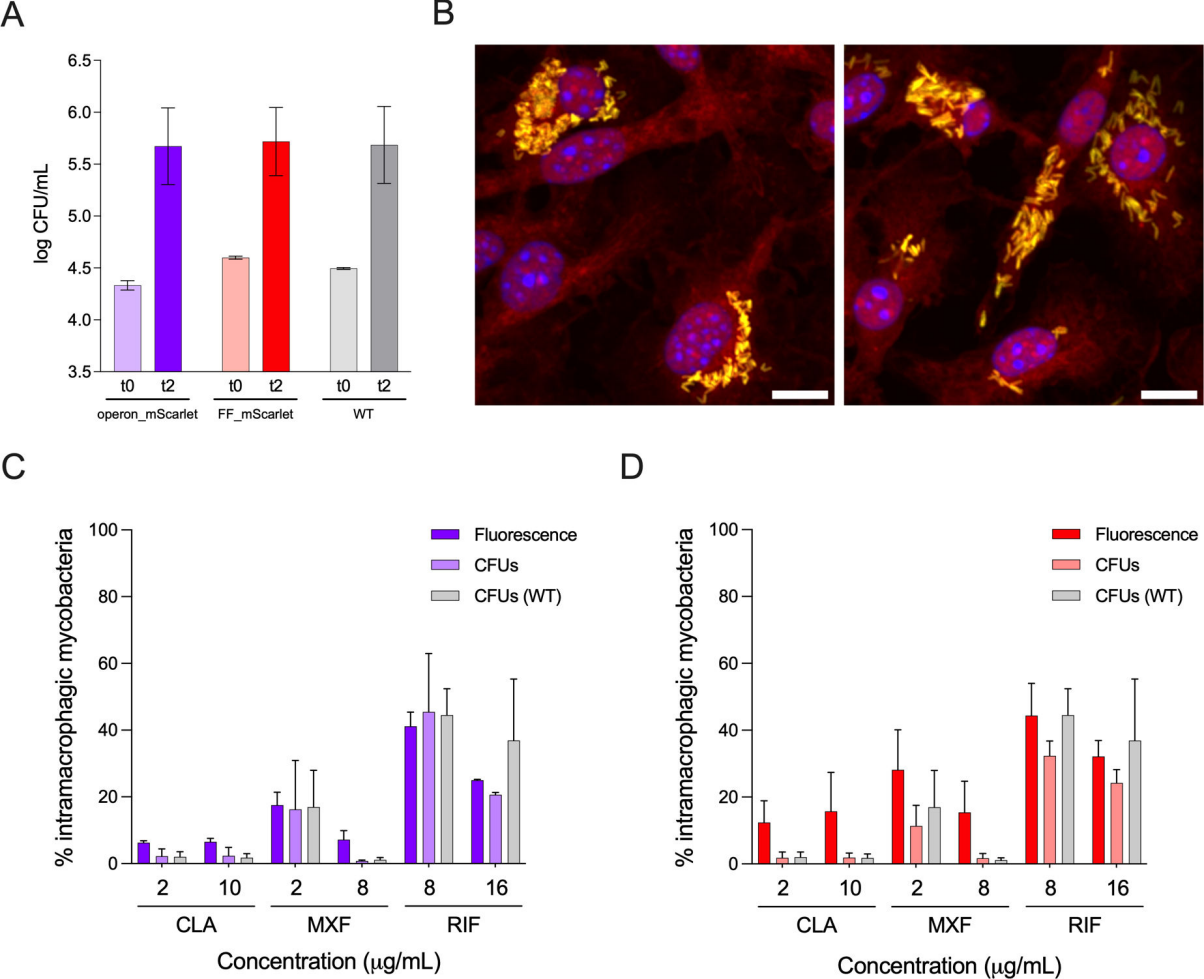
Infection of macrophages by the double-reporter Mab strains. (**A**) Intracellular bacterial load immediately after infection (**t0**) and 2 days after infection (**t2**) with Mab operon_mScarlet (purple), Mab FF_mScarlet (red), or Mab WT (gray) by CFU counting. The graph shows the average ± standard deviations of two to eight independent experiments. (**B**) Representative fluorescence microscopy images of macrophages 2 days after being infected with Mab operon_mScarlet (left) or Mab FF_mScarlet (right), in yellow; in blue, nuclei (DAPI); in red, cytoplasm (HCS CellMask); magnification: 63×; scale bar: 10 µm. Intracellular susceptibility of (**C**) Mab operon_mScarlet and (**D**) Mab FF_mScarlet to clarithromycin (CLA), moxifloxacin (MXF), and rifampicin (RIF), determined either by the mScarlet fluorescent signal or by CFU assay, in comparison to Mab WT by CFU. The graphs show the percentage of bacterial load in treated macrophages compared to nontreated macrophages 2 days post-infection and treatment, expressed as the average + standard deviations of two to three independent experiments.

To evaluate the suitability of the double-reporter strains to infect host cells, murine bone marrow-derived macrophages were infected with the three Mab strains. Figure 6A indicates that all strains grow similarly inside macrophages, having similar intracellular bacterial load after 2 days of infection. Moreover, the expression of mScarlet by the double-reporter strains allows us to monitor the infection by fluorescence microscopy, as seen in Fig. 6B. The images were acquired using an automated confocal microscope (Opera Phenix Plus, PerkinElmer) that also quantifies the mScarlet fluorescent signal inside the host cells.

This quantification was used to evaluate the effect of different antimycobacterial agents (Fig. 6C and D). mScarlet fluorescence showed to be a reliable measure of intramacrophagic bacterial load, allowing the detection of active antibiotics. The magnitude of the antibiotic effect evaluated by the fluorescent signal of the double-reporter strains was overall similar to that assessed by CFU. Interestingly, especially in the case of Mab FF_mScarlet, the apparent effect of the antibiotic was lower when measured by fluorescence. This can be related to the fact that mScarlet is still present in bacteria that are not viable and cannot form colonies. Nonetheless, the transformed strains maintain the same susceptibility profile to the antibiotics as Mab WT in the intra-macrophagic assay.

### The luminescent signal of Mab FF_mScarlet is maintained during *Galleria mellonella* infection

To understand if the new double-reporter Mab strains can be used to monitor infection *in vivo*, we first measured the luminescent signal emitted by the three Mab strains using an *in vivo* imaging system. Only Mab FF_mScarlet emitted a quantifiable luminescent signal in the IVIS system, and the signal correlated with increased bacterial density (Fig. S3). In agreement with what was observed *in vitro* (Fig. 3D), the luminescence of Mab operon_mScarlet in these settings was very low, and it did not correlate with bacterial density, being similar to Mab WT (Fig. S3B). Taking this into consideration, for *in vivo* studies, we only proceeded with Mab FF_mScarlet.

**Fig 7:**
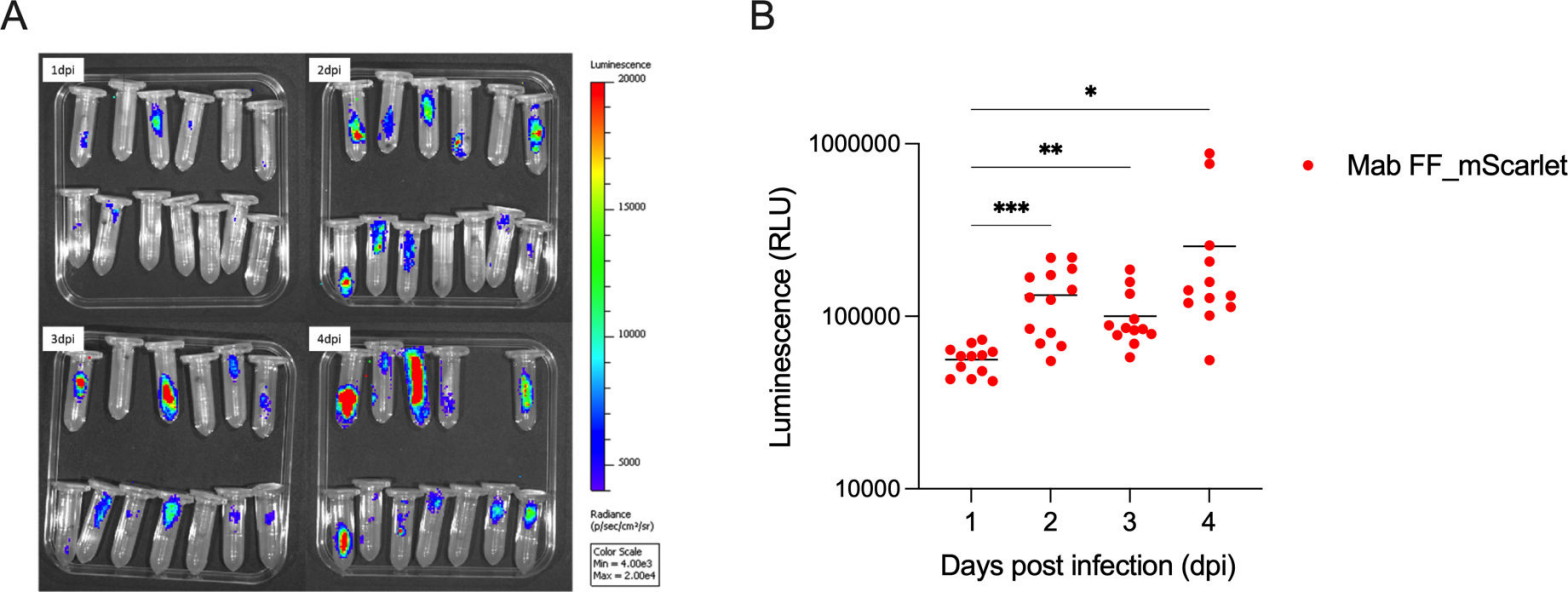
Mab FF_mScarlet can infect *G. mellonella,* and the infection can be followed in real-time using the luminescence emitted by the bacteria. Thirteen *G. mellonella* larvae were infected with 5 × 10^4^ CFU of Mab FF_mScarlet and incubated for 96 h at 37°C. Infection inside the larvae was (**A**) imaged and (**B**) quantified every 24 h in an IVIS Spectrum *In vivo* Imaging system. Statistical analysis was obtained with the mixed-effect analysis using Dunnett’s multiple comparisons in GraphPad Prism 9. *P < 0.05, **P < 0.01, ***P  <  0.001.

To further characterize the Mab FF_mScarlet strain and its ability to infect organisms while maintaining a proper luminescent signal, Mab FF_mScarlet was used to infect *Galleria mellonella* larvae, an established *in vivo* model for Mab (33). Thirteen larvae were infected with 5 × 10^4^ CFU, incubated at 37°C for 96 h, and imaged in IVIS every 24 h. The results in Fig. 7 show that Mab FF_mScarlet can infect *G. mellonella* larvae; luminescence can be detected inside the larvae, and the signal is maintained throughout the infection (Fig. 7A). Moreover, a clear tendency for infection progression was established (Fig. 7B), with the luminescent signal inside the larvae significantly increasing over time.

Altogether, these findings indicate that the double-reporter Mab strains reliably and accurately reflect the activity of antibiotics on Mab, maintaining the same ground characteristics as the non-transformed Mab strain but enhancing its potential for high-throughput screening of thousands of compounds very quickly, even in intracellular assays. Besides, we observed that one of the double-reporter strains can be used for monitoring the infection *in vivo*, making it an important asset in a later phase of the drug discovery pipeline.

## DISCUSSION

The main goal of this work was to develop a tool that can aid high-throughput drug screening for Mab. One of the caveats of current drug screening in mycobacteria is the time it takes to assess the drug’s effect and the difficulty of screening hundreds to thousands of drugs in the same assay, resorting to conventional techniques such as CFU counting. Using reporter strains where the bacterial load and viability can be evaluated in real-time by intrinsic parameters is highly advantageous. We constructed two double-reporter strains of Mab—Mab operon_mScarlet and Mab FF_mScarlet—capable of emitting fluorescence and luminescence. Here, we show that these double-reporter strains can be valuable tools for drug screening *in vitro* and *in vivo*.

Despite an apparent rise in interest, the development of reporter strains in Mab is in its early stages compared to their use in other mycobacterial species. Previous studies, however, can provide valuable clues for advancing the use of reporter strains in Mab. For instance, Gupta et al. (9) performed *in vitro* high-throughput screenings using fluorescent and bioluminescent reporter Mab strains using two distinct plasmid systems. The first contained a fluorescent readout, mCherry, expressed from an episomal vector, and the second relied on an Integration-proficient vector pMV306hsp expressing the entire *luxCDABE* operon [Addgene #26159, (34)]. The authors concluded that both systems were suited for compound evaluation studies though mCherry was hampered by its limited sensitivity and dynamic range (9). The use of reporter strains in macrophage infection assays, which have higher clinical efficacy predictability, is more well-established (35, 36). However, older-generation fluorescent proteins expressed from episomal or integrase-proficient vectors were used. While using eGFP and mCherry is still common, a wide variety of fluorescent proteins are available for constructing fluorescent reporter strains. In their extensive evaluation of different fluorescent proteins and promotor regions in *M. tuberculosis*, Kolbe et al. (20) concluded that more recently developed fluorescent proteins such as mScarlet, which is expressed from a new synthetic promotor element (Pleft*), outperformed both eGFP and mCherry. Therefore, we opted for the red fluorescent protein mScarlet as our fluorescent readout (Fig. 1A and B).

The development of reporter-based assays strongly depends on high and robust reporter protein levels. Reporter gene expression levels and signal intensity are influenced by several factors, such as the type and codon usage of the reporter protein, the type of promotor regions, and the vector systems used to create the transgenic bacterial strains. Consequently, Andreu et al. evaluated the use of different luciferases in *M. tuberculosis* and *M. smegmatis* and concluded that a red-shifted thermostable variant of firefly luciferase was the most well-suited for both *in vitro* and *in vivo* purposes (34, 37). Therefore, our Mab FF_mScarlet expresses that firefly (FF) luciferase, codon-optimized by increasing the GC content and combined with an improved mega Shine Dalgarno sequence (megaSD) under the PG13 promotor control (Fig. 1B). These characteristics have demonstrated the highest bioluminescent signal compared to several other combinations (34, 37).

Finally, the type of vector system strongly influences the stable expression of reporter genes. Commonly applied episomal vectors or integration-proficient vectors have considerable limitations. Several studies indicated that the expression of heterologous genes using replicative vectors is less stable than integrating vectors, making them less suitable for long-term studies (20, 34, 38–40). Yet even integration-proficient vectors are not stably maintained and are easily lost during subculturing without antibiotic selection. However, this can be prevented by using a suicide plasmid providing integrase in *trans* (28, 37). Our engineered plasmids were derived from the L5-based integrase-free integrating plasmid pMV306DIhsp + LuxG13 (37). Using an integrase-free integrative plasmid system circumvents the shortcomings of episomal and integration-proficient plasmids (20, 28, 37, 40). To adapt the plasmid systems for use in Mab, we replaced the kanamycin resistance marker with a more efficient zeocin resistance marker (BleoR) (41) (Fig. 1A and B). In addition, including bi-directional intrinsic terminators in the integration-deficient vectors is advised to protect the expression cassette and the surrounding genome from transcriptional interference (19, 42). Therefore, we equipped both plasmid systems with bi-directional terminators (ttsbiA and ttsbiB) (Fig. 1A and B).

We transformed a laboratory strain of Mab (ATCC 19977) with either pMV306DIhsp + LuxG13+mScarlet or pMV306DIG13 + FFrtCO + mScarlet, generating the double-reporter strains Mab operon_mScarlet and Mab FF_mScarlet, respectively. We confirmed that these strains are suitable for a range of drug screening assays. Both our new double-reporter strains keep the expression of mScarlet in liquid culture for several passages (Fig. 2), indicating that the inserted genetic information, which includes the luminescent genes, is well integrated into the bacterial genome and is maintained from generation to generation. The luminescent signal emitted by Mab FF_mScarlet is stable for at least 30 min in liquid culture after adding the substrate (Fig. S2B), and the same is observed for the autoluminescent strain Mab operon_mScarlet, without the addition of a substrate (Fig. S2A). This indicates that luminescence can be used to assess bacterial viability at any time during an assay using Mab operon_mScarlet and for a considerable amount of time after adding 0.5 mg/mL of D-luciferin when using Mab FF_mScarlet.

We also confirmed that the resistance/selection gene, in this case, zeocin, did not lead to cross-resistance to other antibiotics or new drugs being screened. In fact, we showed that the transformation was stable in the absence of the selecting antibiotic and did not change the growth profile, antibiotic susceptibility, or macrophage infection capability of the original bacteria (designated as Mab WT).

The fluorescence of the two double-reporter strains is stable over time (Fig. 2), even in zeocin-free liquid cultures. Furthermore, the growth curves of the three Mab strains are also independent of zeocin. Therefore, the lack of the selection antibiotic does not impair the growth of the double-reporter strains, nor does it affect fluorescence (Fig. 3C) and luminescence (Fig. 3D) emission, with both signals reaching very high relative intensity values. Besides evaluating the interference caused by the insertion of a selection antibiotic, these results also show that introducing new genetic information in the bacterial genome did not affect the growth profile of the strains. Most importantly, we show an excellent correlation between CFU counting, the reference and most reliable conventional method, and fluorescence and luminescence. That correlation is less strong for the luminescence of Mab operon_mScarlet, which coincides with a decrease in signal after day 3 of the growth curve. As the luminescence of this strain is independent of the addition of a substrate, we can hypothesize that after 3 days in batch culture, the bacteria reduce their production of the bacterial luciferase, encoded by the genes *luxAB* and/or of its long-chain aldehyde substrate, encoded by *luxCDE*. This hypothesis requires future experimental confirmation. That is different for Mab FF_mScarlet, as we add the substrate, D-luciferin, to the bacterial culture right before measuring the luminescent signal, which boosts light emission in that period.

Importantly, double-reporter strains maintained the same antibiotic susceptibility profile as the non-transformed strain, using luminescence as the readout for bacterial viability (Fig. 5). We tested four clinically approved antimycobacterial agents: clarithromycin, a macrolide; amikacin, an aminoglycoside; and linezolid, an oxazolidinone, which are all protein synthesis inhibitors used in multidrug therapies against Mab (43). We also tested moxifloxacin, a fluoroquinolone that inhibits DNA replication, used in the clinic to treat other NTM (43). These results also show that although being resistant to zeocin, the double-reporter strains do not become resistant to other antibiotics, as the MICs obtained for each antibiotic are in line with the MICs for Mab WT (Table 1) and with published values for Mab isolates (44, 45). The luminescent or fluorescent values can be transformed into a drug-response curve, allowing the calculation of the IC_99_ instead of obtaining the MIC by turbidity assessment, which is subjected to person-to-person and assay-to-assay variability (14). This variability is visible in the MIC interval obtained within the independent experiments (Table 1). Furthermore, for Mab WT, it was not possible to calculate the IC_99_ for linezolid and clarithromycin using the resazurin/resorufin fluorescence. On the other hand, the IC_99_ values calculated by luminescence (double-reporter strains) correlated better with the respective MIC values, which suggests that luminescence is an accurate and sensitive readout of bacterial viability and a great alternative to conventional methods.

To support the use of the double-reporter strains’ luminescence as a tool for drug screening in a high-throughput context, we evaluated how different the luminescent signals emitted by a high and a low number of bacteria are, which would allow us to distinguish non-active from active molecules with high precision. Thus, we calculated the *Z*′-factor as a measure of statistical effect size by comparing the luminescent signal of non-treated Mab and Mab exposed to a lethal dose of clarithromycin (Table 2). The calculated *Z*′-factor values for the double-reporter strains indicate that the assay is excellent and suitable for a full-scale high-throughput screening (31). Additionally, these values are higher than the *Z*′-factor obtained for Mab WT using a resazurin assay, reinforcing luminescence as a drug susceptibility screening readout.

To further characterize the double-reporter strains in a more complex and physiological setting, we infected macrophages, one of the main host cells of mycobacteria and the first line of defense against these pathogens upon inhalation (46), with the three Mab strains. We observed that all strains infected bone marrow-derived macrophages, and there were no differences in intracellular bacterial load after 2 days of infection (Fig. 6A). The CFU values (around 10^5^–10^6^ CFU/mL) align with those obtained by another study with the non-transformed Mab strain (47). The fact that the double-reporter strains express fluorescence was essential to observe the mycobacteria inside the macrophages by fluorescence microscopy (Fig. 6B). Taking advantage of the automated high-content screening microscope, Opera Phenix Plus (PerkinElmer), we could quantify the fluorescence signal emitted by the bacteria and test if this was a reliable measure of the intramacrophagic bacterial load. There were no statistically significant differences between the bacterial load quantified by fluorescence and CFU for both double-reporter strains (Fig. 6C and D). Besides, there were no differences between the CFU quantification for the double-reporter and the WT strains. These results indicate that the transformation did not alter the intracellular antibiotic susceptibility of the bacteria and that the double-reporter’s fluorescence can be used to quantify the bacterial load inside host cells.

Luminescence could be used to evaluate the viability of intracellular bacteria (48, 49). Antibiotics can have bacteriostatic activity, stalling the bacteria’s growth without eliminating them, as described for clarithromycin against NTM (50, 51). Therefore, a non-viable bacillus can still emit fluorescence through the presence of mScarlet but would not emit light. However, the sensitivity of luminescent plate readers is much lower than that of an automated fluorescent microscope. Moreover, the number of bacteria in intracellular assays is usually not high enough to be detected in a microplate reader (results not shown). Therefore, in the experimental settings, we have developed, fluorescence microscopy is the better strategy for monitoring intracellular infection in either fixed or live cells.

Once the double-reporter strains were validated for *in vitro* drug screening, it was important to investigate if we could take advantage of their reporter characteristics *in vivo*. We chose the *G. mellonella* infection model as it is a simple, low-cost, reproducible model, already established for Mab infection (33), and uses luminescence as readout by IVIS imaging. Bioimaging reduces the number of larvae per experiment, as the same animals are repeatedly imaged instead of euthanized at each time-point, thus allowing us to monitor the infection’s progression over time and obtain more reliable results. We infected the *G. mellonella* with Mab FF_mScarlet, as we observed that the luminescent signal emitted by Mab operon_mScarlet was not intense enough to be detected with this equipment (Fig. S3). Indeed, expressing an improved firefly luciferase, Mab FF_mScarlet has the advantage of having an emission spectrum shift towards the red (maximum at 620 nm) in comparison with reporters that express other luciferases, which emit at lower wavelengths (37). Mab operon_mScarlet expresses the whole lux operon that produces light in the blue range (490 nm), which is more susceptible to tissue absorption and scattering (34, 52). We proceeded only with Mab FF_mScarlet although it requires a D-luciferin injection before imaging. Our results (Fig. 7) show that the luminescent signal is detected inside the larvae and that the signal increases with the progression of the infection, indicating that this reporter strain can also be used to monitor Mab infection *in vivo*. The *G. mellonella* infection model was also validated for drug susceptibility testing (33). Therefore, its infection with Mab FF_mScarlet can be used as a tool to select new molecules before being tested in more complex and expensive animal models. The fluorescence valence of our double-reporter strains, due to its high intensity conferred by mScarlet being controlled by the promoter Pleft, although being difficult to detect through animal tissue, can be used to visualize the individual bacilli in slices of infected lungs or by flow cytometry, as shown by Kolbe et al. (20).

Overall, this study shows the potential of new double-reporter Mab strains as tools to help discover new compounds with activity against an increasingly prevalent pathogen whose unpredictability and intrinsic antimicrobial resistance raise serious concerns. The major advantage of these double-reporters is the extensive range of experimental settings in which they can be used, in opposition to single-reporter bacteria. In some situations, luminescence is important to gain insight into the metabolic state of the bacteria, an aspect especially relevant for mycobacteria, as they use latency to hide from the host defenses. However, recent advances in fluorescence microscopy, like the development of high-content microscopes, make the ability to express fluorescence essential for initial screenings with thousands of compounds. Therefore, depending on each scientific question, we can use the same bacterial strain and choose the readout that better answers it. Also, sometimes, it can be useful to work with a strain like Mab FF_mScarlet, which expresses a very intense luminescent signal boosted by the addition of a substrate, but in other cases, it can be more practical and as informative to measure Mab operon_mScarlet’s autoluminescence, which allows the evaluation of the bacterial load in a non-invasive way without any assay manipulation. There are still several experimental models in which these strains can be applied that we did not explore in this study, like infection of other types of host cells and more complex contexts, as in three-dimensional models such as spheroids, granulomas, or organoids. It would also be interesting to explore the use of these strains for the study of biofilm formation and for the establishment of a model of drug screening in that setting. Importantly, the constructs with which we transformed Mab can be used to transform any other mycobacterial species, creating double-reporters of laboratory strains or clinical isolates, which opens new avenues for host-directed therapies.

## SUPPLEMENTAL MATERIAL

Supplemental material spectrum.00362-24-s0001.pdf Supplemental figures and tables.

ASM does not own the copyrights to Supplemental Material that may be linked to, or accessed through, an article. The authors have granted ASM a non-exclusive, world-wide license to publish the Supplemental Material files. Please contact the corresponding author directly for reuse.
