# Supplementary material for: Characterization of novel double-reporter strains of Mycobacterium abscessus for drug discovery: a study in mScarlet
Source: Microbiol Spectr. 2024 Aug 27;12(10):e00362-24. doi: 10.1128/spectrum.00362-24 (PMC11448253; doi:10.1128/spectrum.00362-24)
Supplement: Supplemental material — Supplemental figures and tables. [file spectrum.00362-24-s0001.pdf]

Characterization of novel double-reporter strains of *Mycobacterium abscessus* for drug discovery: a study in mScarlet

Clara M. Bento,[a,b,c] Kevin van Calster,[e]* Tatiana Piller,[e] Gabriel S. Oliveira, [a,d] Linda de Vooght,[e] Davie Cappoen,[e]* Paul Cos,[e] M. Salomé Gomes,[a,d] Tânia Silva[a,d]#

[a]i3S – Instituto de Investigação e Inovação e Saúde, Universidade do Porto, Porto, Portugal

[b]IBMC – Instituto de Biologia Celular e Molecular, Universidade do Porto, Porto, Portugal

[c]Programa Doutoral em Biologia Molecular e Celular (MCBiology), Instituto de Ciências Biomédicas Abel Salazar da Universidade do Porto, Porto, Portugal

[d]ICBAS – Instituto de Ciências Biomédicas Abel Salazar da Universidade do Porto, Porto, Portugal

[e]Laboratory for Microbiology, Parasitology and Hygiene (LMPH), Wilrijk, Belgium

#Address correspondence to Tânia Silva, tania.silva@i3s.up.pt

*Present address: Kevin van Calster – Department of Infectious Diseases in Humans, Immune Response, Sciensano, Brussels, Belgium; Davie Cappoen – Service of Risk & Health Impact Assessment, Institute for Public Health, Sciensano, Belgium.

Supplemental material

Table S1. List of plasmids, antibiotic resistance markers (Amp – ampicillin; Kan – kanamycin; Zeo – zeocin; Hyg – hygromycin B), and respective sources used in this study.

Plasmid	Description	Resistance marker	Source
pMV306DIhsp+LuxG13	L5 integrase-deficient integrating plasmid encoding Bacterial Luciferase operon (LuxG13)	Kan^r	Addgene (Plasmid #49999)
pKM496	Plasmid used to amplify the EM7 promoter and Zeocin resistance gene	Zeo^r	Addgene (Plasmid #109301)
pML1357	Plasmid used to amplify the bi-directional terminators (ttsbiA and ttsbiB)	Hyg^r	Addgene (Plasmid #32378)
L5 attB::Pleft* mScarlet	Integrating plasmid encoding the fluorescent protein, mScarlet under control of the Pleft* promoter	Kan^r	Addgene (Plasmid #169410)
pMV306DIhsp+LuxG13+mScarlet	L5 integrase-deficient integrating plasmid containing both the bacterial Luciferase operon (LuxG13) and the mScarlet gene	Zeo^r	This study
pUC57:FFrtCO	Plasmid containing the gene encoding for a thermostable, red light emitting firefly luciferase, codon optimized for expression in mycobacteria	Amp^r	Genscript

pMV306DIG13+FFrtCO+mScarlet	L5 integrase-deficient integrating plasmid containing genes encoding a codon-optimized, thermostable, red-shifted Firefly luciferase and fluorescent protein mScarlet	Zeo^{r}	This study
pBS-Int	Suicide plasmid expressing the L5 integrase	Amp^{R}	Addgene (#50000)

Table S2. Primers used in this study. Underlined sequences are overlapping ends for HiFi assembly.

Target	Template	Primer Name	Primer
EM7::BleoR	pKM496	EM7::BleoR.FOR	5'-tgccgttcccgccaggttgacaattaatcatcggcatagtatatcgg -3'
		EM7::BleoR.REV	5'-ggtatctagatgcctggcagttccctac-3'
ttsbiB	pML1357	ttsbiB.FOR	5'-gccaggcatctagatacctgaaaaaaaaaaagcgccgcag-3'
		ttsbiB.REV	5'-agtggaactagtaaaaaaaaaagcgccgcaactgcg-3'
Pleft* mScarlet	L5 attB::Pleft*mScarlet	Pleft* mScarlet.FOR	5'-gccgcgggagcgcaacgcgtgc-3'
		Pleft* mScarlet.REV	5'-gctaaagctttcgatcgtacgctagttaactacgt -3'
ttsbiA	pML1357	ttsbiA.FOR	5'-tacgatcgaaagctttagctaattaattggggaccc-3'
		ttsbiA.REV	5'-cggctctagctaaaaaaaaaagccccgcgattgcgggg-3'
Linearized 1	pMV306DIhsp+LuxG13_zeo	pmv306DIhspLuxG13zeo.FOR	5'-tttttttttagctagagccgtgaacgac -3'
		pmv306DIhspLuxG13zeo.REV	5'-ttgcgctcccgcggccatgatggc -3'
Linearized 2	pMV306DIhsp+LuxG13+mScarlet	pMV306DIhspmScarlet.FOR	5'-gtaagtcgacgtagttaactagcgtacgatcgac-3'
		pMV306DIhspmScarlet.REV	5'-ggcgatcgcacgcgttgcgctcg-3'
Pg13	pMV306DIhsp+LuxG13	Pg13.FOR	5'-acgcgtgcgatcgccactagcgcc-3'
		Pg13.REV	5'-cttctgaattctcggttaccaagcgtgca-3'
megaSD + FFrtCO	pUC57:FFrtCO	megaSD + FFrtCO.FOR	5'-ggtaaccgagaattcagaaggagaagtaccgatggag-3'
		megaSD + FFrtCO.FOR	5'- agttaactacgtcgacttacagcttcgacttgc-3'

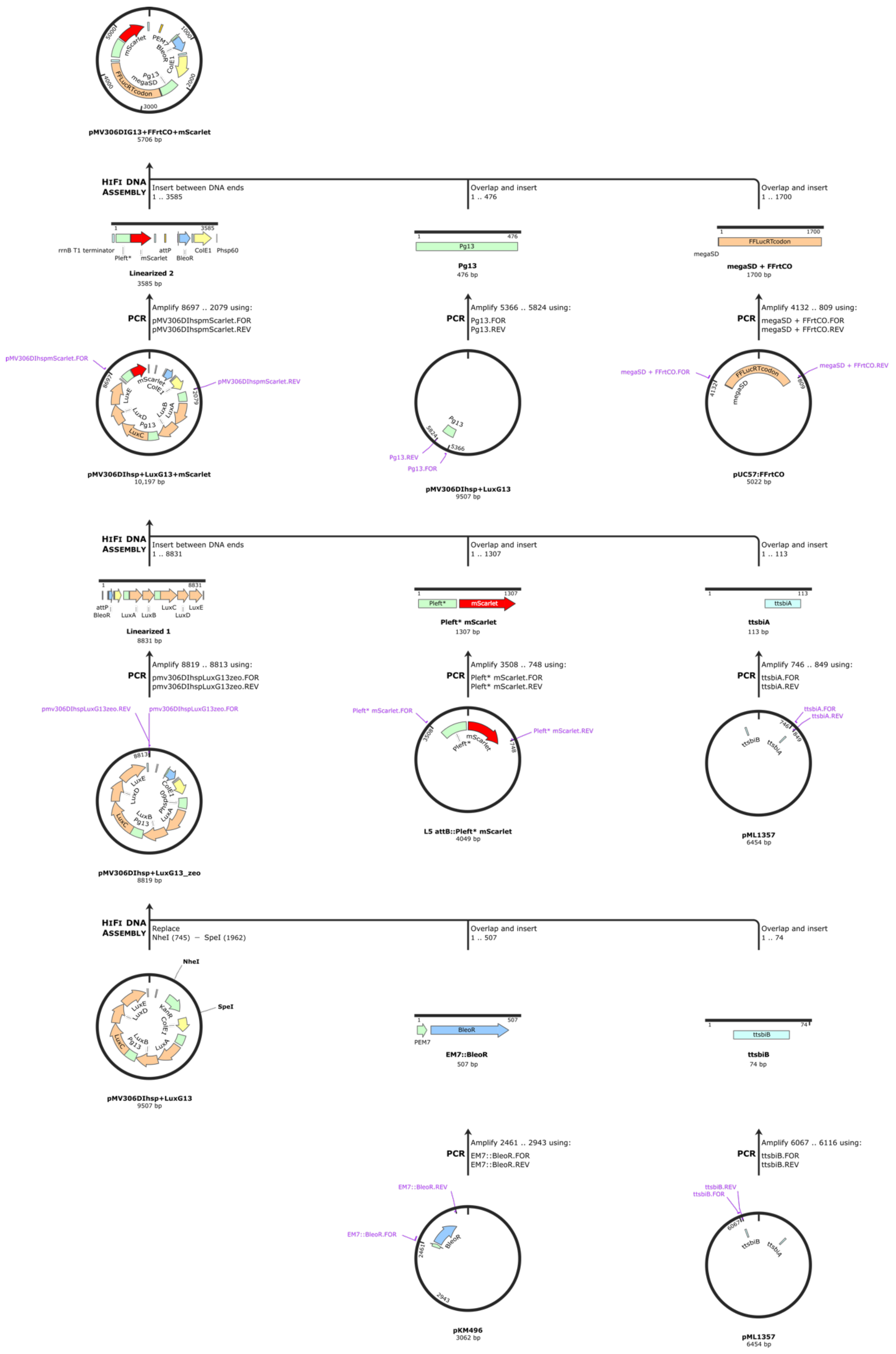

Figure S1. Schematic representation of the cloning strategy used for constructing the integrating reporter plasmids pMV306DIhsp+LuxG13+mScarlet and pMV306DIG13+FFrtCO+mScarlet.

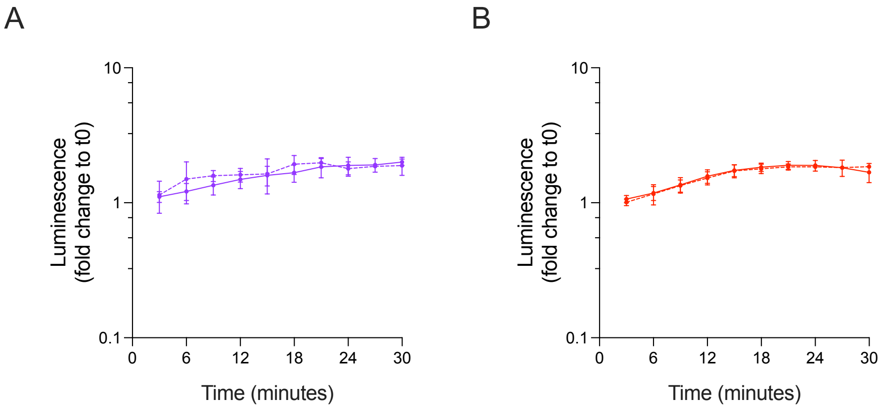

Figure S2. The luminescent signal was measured in 3-minute intervals of **(A)** Mab operon_mScarlet cultures after the addition of D-luciferin at 0.5 mg/mL (10% v/v) (t=0) and **(B)** Mab FF_mScarlet cultures. The dashed line represents a bacterial suspension 10 times more diluted than the one represented by the full line. The results are expressed as fold change to RLU at t=0. The symbols represent the average ± standard deviations of two independent experiments.

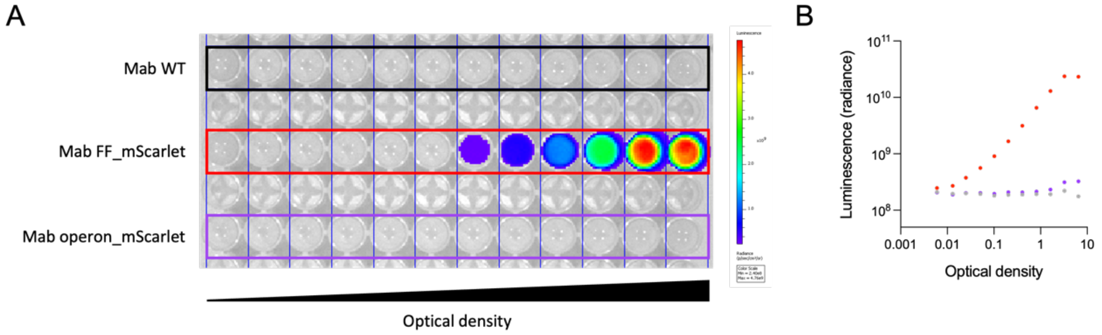

Figure S3. The luminescent signal of the three Mab strains was detected using an IVIS® Spectrum In Vivo Imaging system (PerkinElmer). **(A)** Bacterial suspensions were successively diluted 1:2 in a 96-well plate, and the luminescent signal was measured after exposing 0.5 seconds. The luminescence expressed in radiance by Mab WT (grey), Mab FF_mScarlet (red), and Mab operon_mScarlet (purple) are plotted in **(B)** and were analyzed with the Living Image v4.3.1 software.
